# Isolated tricuspid valve surgery; long-term outcomes based on Tehran Heart Center data bank report

**DOI:** 10.1186/s13019-021-01394-1

**Published:** 2021-02-23

**Authors:** Seyed Hosssein Ahmadi Tafti, Farshid Alaeddini, Mahmood Shirzad, Jamshid Bagheri, Abbas Salehi Omran, Mehrdad Mahalleh, Shiva Shoja, Negar Omidi

**Affiliations:** 1 Tehran Heart Center, Tehran University of Medical Sciences, Tehran, Iran; 2 Cardiovascular Research Center, Tehran University of Medical Sciences, Tehran, Iran; 3 Al-Zahra Hospital, Isfahan University of Medical Sciences, Isfahan, Iran

**Keywords:** Tricuspid valve, Valve repair/replacement, In-hospital mortality

## Abstract

**Background:**

Given that isolated tricuspid valve (TV) repair or replacement is performed relatively rarely, we sought to evaluate the rate of long-term mortality and readmission following this surgery.

**Methods:**

The current study was conducted in Tehran Heart Center on patients who underwent isolated TV repair or replacement between 2010 and 2018. Totally, 197 patients (repair = 150 vs replacement = 47) were included in our study and were then followed right after surgery for a median of 8 years to assess the incidence of postoperative events, readmission, and all-cause mortality.

**Results:**

The final analysis was conducted on 197 patients at a mean age of 44.4 ± 13.8 years. Most of the patients were female (56.9%). Ejection fraction, TAPSE, and right ventricular function improved in both groups after TV surgery. Length of stay in the intensive care unit per hour and hospitalization per day were higher in the replacement group and compared to the repair group (158.34 vs. 55.11 and 18.21 vs. 9.34, respectively). In-hospital mortality occurred in 20 patients, of whom 15 had TV replacement. Readmission occurred in five (2.5%) patients,all were in the repair group.

**Conclusions:**

The result of this single-center study showed that TV replacement is associated with a higher rate of postoperative events and all-cause mortality compared to TV repair. Whereas, repair group had a higher rate of readmission. Therefore, the overwhelming tendency is toward repair; nonetheless, no hesitation is permissible if a replacement is adjudged to confer a better outcome for the patient.

## Introduction

Tricuspid valve (TV) disease is far less common than is mitral or aortic valve involvement, and it is often ignored because its regurgitation or stenosis can be well tolerated [1–3]. TV regurgitation that leads to surgery can be either organic or secondary. The etiologies of organic TV disease include rheumatic fever, tumors such as myxomas and carcinoids, genetic diseases such as Marfan’s disorder, and congenital diseases such as Ebstein’s anomaly. Secondary causes are mostly due to left-heart pathologies such as left-side heart failure, progressive biventricular dysfunction, and pulmonary hypertension. The severity of TV dysfunction tends to rise steadily, and it is mostly diagnosed when patients develop significant symptoms [4]. The majority of patients are controlled with pharmacotherapy until it fails, necessitating their referral to surgeons for decision-making on the repair or replacement of the valve based on the symptoms and severity of the disease. Isolated TV repair is feasible, whereas TV replacement is not frequently performed due to the high rate of short- and long-term morbidity and mortality based on the characteristics of patients and other concomitant disorders [5, 6].

Previous studies have evaluated the short- and long-term outcomes of TV repair or TV replacement in other countries [7–9] but there are limited studies about outcomes of isolated TV surgery in middle income countries. We, therefore, aimed to assess the incidence of the long-term morbidity and mortality of isolated TV repairing by ring annuloplasty or tricuspid replacement with or without atrial septal defect (ASD) closure in Tehran Heart Center.

## Methods

### Study population

This retrospective cohort study was performed using the Tehran Heart Center Valve Surgery Data Bank. All the patients who had TV repair or replacement between 2010 and 2018 whose information was recorded in our database were enroled. The exclusion criteria were concomitant mitral valve replacement and aortic valve replacement and uncompleted recorded data. All the stages, from data collection to data analysis, were performed without revealing the identity of the patients. The study protocol was approved by the institutional ethics committee and research board.

### Follow-up protocol

In keeping with the protocol of the Tehran Heart Center Surgery Data Bank, general practitioners and research nurses interviewed and examined the patients to complete their data on symptoms, major risk factors of cardiovascular disease, and baseline characteristics. The patients were observed and interviewed during hospitalization and for a 30-day period after surgery to record any early in-hospital mortality and readmission. In addition, long-termfollow-up was done to evaluate the echocardiographic indices and incidence of postoperative events among the patients whether they were readmitted to our center or elsewhere.

### Study endpoints

The major endpoints of our study were comprised of 1) determining the incidence of all-causein-hospital mortality, 2) determining the rate of postoperative events and readmission over a 30-day period, and 3) determining the incidence of mortality within 8 years.

### Statistical analysis

Categorical variables are presented as percentage (number) and were evaluated using chi-squared test or Fisher’s exact test. Continuous variables are presented as mean ± standard deviation. Analyses of continuous variables were done using student’s t-test. SPSS 20.0 statistical software (IBM, Chicago, IL, USA) was used for data analysis and *P* ≤ 0.05 was the criterion of significance.

## Results

### Population

The study population was comprised of 197 patients at a mean age of 44.4 ± 13.8 years and a mean body mass index of 24.9 ± 4.4 kg/cm^2^ who underwent TV repair or replacement either solely or with concomitant ASD surgical closure. Women accounted for 56.9% (*n* = 112) of the patients. The median follow-up period was 57.6 months.

TV repair was performed in 150 (76.1%) patients, bioprosthetic valve replacement in 41 (87.23%), and mechanical valve replacement in 6 (12.77%).

All the patients were investigated for postoperative complications, readmission, and 30-dayin-hospital mortality and were included in the final analysis. The baseline characteristics of the study population are summarized in Table 1.

**Table 1:** Baseline characteristics and echocardiography features of the study population

Characteristic	Implant Type		***P***-value
	Repair (***n*** = 150)	Replacement (***n*** = 47)	
**Demographics**			
Age, mean (SD)	43.0 (13.6)	48.8 (13.5)	0.011
Gender (female), n (%)	81 (54.0)	31 (66.0)	0.149
Body mass index, mean (SD)	24.9 (4.4)	24.9 (4.4)	0.931
**Past Medical History and Risk Factors**			
Diabetes, n (%)	6 (4.0)	6 (12.8)	0.039
Hyperlipidemia, n (%)	20 (13.4)	11 (23.4)	0.102
Rheumatic valve, n (%)	30 (20.0)	36 (76.6)	< 0.0001
Atrial septal defect, n (%)	120 (79.5)	8 (17.0)	
**Pre-operation**			
NYHA functional class, mean (SD)	1.75 (0.73)	1.98 (0.74)	
Ejection fraction, mean (SD)	49.74 (5.76)	47.37 (7.83)	0.002
TAPSE, mean (SD)	21.27 (6.93)	17.50 (7.30)	0.005
RVD, mean (SD)	44.09 (8.21)	39.47 (8.87)	0.003
PAP, mean (SD)	39.76 (12.57)	40.21 (12.73)	0.452

*NYHA* New York Heart Association, *TAPSE* Tricuspid annular plane systolic excursion, *RVD* Right ventricular dysfunction, *PAP* Pulmonary arterial pressure

Continuous variables are presented as the mean (SD) or the median (25th and 75th percentiles)

Categorical variables are described as frequencies (percentages); n (%)

Concerning the etiology of TV disease, rheumatic valve etiology was more common in the replacement group than in the repair group (76.6% vs. 20%), whereas ASD was more prevalent in the repair group (79.5% vs. 17%).

### Endpoints

Based on the results of the study, there was an improvement in the left ventricular ejection fraction in both groups postoperatively (Table 2). Furthermore, tricuspid annular plane systolic excursion (TAPSE), which is a marker of right ventricular (RV) function, was improved following surgery (Table 2). Postoperative events occurred in 3.33% of the repair group and 21.27% of the replacement group. The median time interval to the first event was 2.5 (2.12) and 6.3 (3.5) months after surgery in the repair and replacement groups, respectively. Table 2 shows the postoperative echocardiographic outcomes of the study population after surgery. As is shown, the incidence of endocarditis was higher in the repair group than in the replacement group, but the frequency of valve malfunction and redo surgery was higher in the replacement group. Intensive care unit (ICU) hours and the ventilation time were higher in the replacement group and, consequently, the length of hospital stay was also higher in this group (Table 2). Higher total ICU hours were not statistically associated with high preoperative pulmonary arterial pressure (PAP) in the replacement group and the repair group (correlation: 0.275, *P* = 0.082 vs 0.060, *P* = 0.470, respectively), but the association has clinical significance in the replacement group. The rate of all-cause mortality in the replacement group was higher than that in the repair group, (32.6% vs 3.3%, respectively). Only five (3.3%) patients from repair group readmitted to the hospital (Table 2).

**Table 2:** Echocardiographic features and outcomes of the study population

Characteristic	Implant Type		***P*** Value
	Repair (***n*** = 150)	Replacement (***n*** = 47)	
**Post-operation**			
Dyspnea	24 (16.0)	5 (10.6)	0.365
Orthopnea	0 (0.0)	1 (2.1)	0.239
Palpitation	4 (2.7)	2 (4.3)	0.630
Acute chest pain	2 (1.3)	1 (2.1)	0.561
NYHA functional class, mean (SD)	1.05 (0.25)	1.12 (0.42)	0.373
Ejection fraction, mean (SD)	51.12 (5.02)	47.72 (5.50)	0.005
TAPSE, mean (SD)	16.80 (3.52)	16.20 (3.77)	0.422
RVD, mean (SD)	34.80 (6.31)	32.20 (5.33)	0.064
PAP, mean (SD)	32.00 (9.29)	30.66 (8.06)	0.514
ICU, h	55.11 (54.08)	158.34 (286.67)	0.020
Ventilation time	14.91 (30.25)	89.56 (256.44)	0.058
Length of stay, d	9.34 (4.48)	18.21 (16.39)	0.001
**Events**			
Readmission	5 (3.3)	0 (0.0)	0.341
Malfunction/valve thrombosis	2 (1.3)	3 (6.4)	0.089
Redo surgery	0 (0.0)	7 (14.8)	0.000
Hemorrhagic stroke	1 (0.6)	0 (0.0)	1.000
Endocarditis	2 (1.3)	0 (0.0)	1.000
Mortality	5 (3.3)	15 (32.6)	0.000

*NYHA* New York Heart Association, *TAPSE* Tricuspid annular plane systolic excursion, *RVD* Right ventricular dysfunction, *PAP* Pulmonary arterial pressure, *ICU* Intensive care unit

## Discussion

In the present study, we evaluated long-term mortality and morbidity between 2 groups of TV repair and TV replacement over a median of 8 years. We found that mortality was higher (32.6%) in the replacement group than in the repair group (25.9%); nevertheless, this finding should be interpreted in light of the fact that our study sample was small. Consistent with our results, previous studies have also concluded that TV replacement is associated with considerably high mortality compared with TV repair [10, 11]. Guenther et al. evaluated 416 patients over a median of 5.9 years and found that 30-day mortality after TV repair was 13.9% (43/310) opposed to 33% (35/106) after TV replacement (*P* = 0.0001) [10]. In line with it, multiple studies have reported that the in-hospital mortality after TV replacement ranges between 14.5 and 48% [12]. It is crucial for surgeons to select their patients cautiously to reduce the rate of mortality after TV replacement. Topilsky et al. also believed that candidating the right patient for TV replacement might lower the rate of mortality [13].

There are other independent predictors of mortality after isolated TV surgeries such as the New York Heart Association (NYHA) functional class [13, 14] and RV function parameters [13, 15]. These variables should be considered in the decision for the medical or surgical treatment of TV disease. It is apparent that both of them play central roles in both preoperative clinical status and postoperative outcomes [4, 16–18]. As is shown in our results, postoperative RV dysfunction and the NYHA functional class improved in both groups by comparison with the preoperative measurements, and we detected no correlation between either TAPSE or the NYHA functional class and mortality. Bevan et al. [19] and Buzzatti et al. [20] posited that RV dysfunction played an important role in preoperative characteristics such as ascites and also postoperative outcomes. Topilsky et al. reported that a preoperative NYHA functional class of 4 was associated with a higher chance of mortality [13]. In a propensity score study, Calafiore et al. showed that functional benefits were present with moderate-to-severe functional tricuspid regurgitation subjected to mitral surgery if the TV was concomitantly diseased [21].

There are different causes of readmission after TV surgery irrespective of the type of Surgery (repair vs. replacement) including congestive heart failure (CHF), arrhythmia, respiratory and surgical complications, cerebrovascular arrest, infections and acute kidney injury [22]. In our study, only repair group experienced readmission due to CHF, renal failure, transient ischemic attack and valve dysfunction. The patients in replacement group had higher rate of mortality.

PAP is associated with worse outcomes in patients undergoing isolated tricuspid surgery [14]. Likewise, we observed a drop in postoperative PAP in both groups. Preoperative PAP had a significant correlation with the length of stay in the ICU, and it was strongly higher in the RV replacement group. Buzzatti et al. observed a relationship between the presence of a higher PAP leading to poor outcomes and mortality in their patients undergoing TV replacement. Similarly, Mangoni et al. concluded that one of the factors associated with poor outcomes was a high PAP [6]. We believe that the high preoperative PAP in our replacement group might have resulted in the higher rate of mortality in this group.

We have previously demonstrated that redo surgery is a major determinant of early and long-term mortality [3, 5, 23]. In this study, we also observed that none of the patients in the TV repair group had redo surgery, whereas 14% of the TV replacement group patients were scheduled for redo surgery, which can cause a lower rate of survival in a longer follow-up period. Great precaution may be needed when performing redo surgery in patients with isolated TV disease due to the high recurrence of tricuspid regurgitation after redo surgery [23]. Our results also revealed that only 3.5% of the entire study population (*n* = 7, 14.8% in the replacement group) needed redo surgery, which is lower than the rates in previous studies [24–27].

Rheumatic etiology is one of the most important predictors of a poor outcome. This is understandable because rheumatic disease is a destructive and ongoing process causing extensive and sometimes irreparable damage not only to the TV but also to the myocardium [28]. We found that the incidence of rheumatic valve disease was higher in the TV replacement group than in the TV repair group (76.6% vs 20.0%). Moreover, our results revealed a higher mortality rate in the replacement group. Previous studies have also reported that rheumatic heart disease is correlated with mortality in patients after TV surgery [6, 28]. It can be referred from our results that rheumatic valve disease might have caused the high mortality in our TV replacement group compared with our TV repair group.

We had 1 patient with hemorrhagic stroke in the TV repair group who had a history of atrial fibrillation and was on vitamin K antagonist regimen. Postoperative neurological impairment after cardiac surgery is a serious complication. In a recent study, Raffa et al. evaluated the incidence of different types of stroke after a variety of cardiac surgeries and found that the incidence of hemorrhagic stroke was relatively low compared with ischemic stroke and it occurred in patients undergoing concomitant cardiac surgeries (TV surgery concomitant with coronary artery bypass graft or aortic valve surgery) [29].

Based on the past medical history of our patients, most of the ASD cases were in the TV repair group (79.5% vs 17.0%) rather than in the TV replacement group. ASD closure surgery is one of the concomitant procedures in TV surgery [30].

In our study, we found that ICU hours and the length of hospital stay were significantly higher in the replacement group, even though this group comprised fewer patients than did the repair group. Alqahtani et al. evaluated the outcomes of surgical treatment in 45,477 patients, 15% of whom only had isolated tricuspid surgery. They found that TV surgery was associated with a long duration of hospitalization, which is reported to be higher in other types of valve surgeries [31]. In another study, TV replacement and repair propensity-matched cohorts had similar ICU lengths of stay, but the replacement group had a significantly longer hospital length of stay [32].

It is believed that diabetes, the level of creatinine, and the white blood cell count play important roles in mortality insofar as they can increase the chance of postoperative infection and mortality afterward. Among the patients who died in the present study, 3 patients had leukocytosis due to urinary tract infection, which caused septicemia and led to death. This finding is concordant with the results reported by Fowler et al., who reported a higher rate of complications such as longer lengths of hospital stay and higher rates of mortality in patients with major infections after cardiac surgery [33].

In the current study, we had 2 patients with endocarditis as the etiology of repair surgery; both of them expired. (The power of the study made it impossible to evaluate the statistical difference.) Nonetheless, Singh et al. reported that the organic pathology of the valve could significantly increase the occurrence of mortality in TV replacement as opposed to TV repair irrespective of the type of implant. Pfannmüller et al. concluded that mortality could be higher if surgery was performed under urgent conditions due to endocarditis or other cardiac diseases [3]. The discrepancy between the results of the present study and those previously reported might be due to our small sample size. We, thus, recommend further evaluations on larger sample volumes.

### Study limitations

We did not calculate the predictive score of mortality (eg, the EuroSCORE), nor did we assess the reoccurrence rate of postoperative regurgitation. In addition, the study population was relatively small and we were unable to perform a long-termfollow-up on our patients. Moreover, there were differences in the some of the baseline characteristics of the study population. Another salient weakness of our study is our failure to record information concerning the presence of ascites and anasarca, which have major associations with mortality. It must be mentioned, due to low percentage of mortality, evaluating the predictors of the mortality in either groups, was not the aim of present study.

## Conclusions

This retrospective study of isolated TV surgery, conducted in a single center over nearly a decade, showed that replacement, by comparison with repair, was associated with a high rate of postoperative events, mortality, and lower readmission, which confirms why it is performed rarely. Further studies with larger sample size are required.

## Data Availability

The datasets used and/or analysed during the current study are available from the corresponding author on reasonable request.

## Abbreviations

ASD: Atrial septal defect
CHF: Congestive heart failure
ICU: Intensive care unit
NYHA: New York Heart Association
PAP: Pulmonary arterial pressure
RV: Right ventricle
TAPSE: Tricuspid annular plane systolic excursion
TV: Tricuspid valve
